# What we have changed our minds about: Part 1. Borderline personality disorder as a limitation of resilience

**DOI:** 10.1186/s40479-017-0061-9

**Published:** 2017-04-11

**Authors:** Peter Fonagy, Patrick Luyten, Elizabeth Allison, Chloe Campbell

**Affiliations:** 1 Research Department of Clinical, Educational and Health Psychology, University College London, London, UK; 2 Faculty of Psychology and Educational Sciences, KU Leuven, Leuven, Belgium

**Keywords:** Borderline personality disorder, Resilience, Epistemic trust, Mentalizing, Attachment, Psychopathology

## Abstract

This paper sets out a recent transition in our thinking in relation to psychopathology associated with personality disorder, in an approach that integrates our thinking about attachment, mentalizing (understanding ourselves and others in terms of intentional mental states) and epistemic trust (openness to the reception of social communication that is personally relevant and of generalizable significance) with recent findings on the structure of both adult and child psychopathology and resilience. In this paper – the first of two parts – we review evidence suggesting that a general psychopathology or p factor underlies vulnerability for psychopathology. We link this p factor to a lack of resilience using Kalisch and colleagues’ positive appraisal style theory of resilience (PASTOR). We argue that vulnerability for (severe) psychopathology results from impairments in three central mechanisms underlying resilience – positive situation classification, retrospective reappraisal of threat, and inhibition of retraumatizing triggers – which in turn result from a lack of flexibility in terms of social communicative processes. We suggest that, from this perspective, personality disorders, and borderline personality disorder (BPD) in particular, can be considered to be the prototype of disorders characterized by a lack of resilience. Part 2 proposes an evolutionary developmental psychopathology account linking this inflexibility in social communication to problems with the development of epistemic trust – that is, an evolutionary pre-wired social communication system that normally facilitates resilience through salutogenesis, that is, the capacity to learn and derive benefit from the (social) environment.

## Background

A challenge for contemporary thinking about psychopathology arises from a general neglect by adult psychopathologists of the developmental psychopathology tradition established by Sroufe and Rutter [1] over 30 years ago. Specifically, the fact that when we consider an individual’s psychiatric history over their life course, it rarely follows the discrete, symptom-led and time-limited categories that traditional models have used in conceptualizing mental disorder. This has increasingly come to be regarded as constituting a slow-burning crisis in the way we understand, and by extension treat, mental disorders. There is a heightened recognition of the salience of transdiagnostic features in clinical presentations as well as across treatment protocols [2, 3]. Particularly in cases of more severe and persistent mental health difficulties, an individual’s clinical presentation changes over time, one typical example being progression from conduct disorder to depression [4], or the extensive comorbidity between traditional ‘symptom’ disorders and personality disorders (PDs) (e.g. [5]).

Here we posit a reconceptualization of psychopathology associated with PD that speaks to these conceptual and diagnostic enigmas, in an approach that integrates our thinking about mentalizing (i.e. understanding ourselves and others in terms of intentional mental states) and epistemic trust (i.e. openness to the reception of social communication that is personally relevant and of generalizable significance) with recent findings on the structure of both adult and child psychopathology [3] and resilience [6].

At the core of the thinking set out here is an emphasis on the relationship between the social environment as a system on the one hand, and individual differences in the capacity for social cognition (as defined below) on the other. We argue that the presence or absence of resilience is the outcome of the dynamics of this relationship. Understanding the nature of resilience, we suggest, requires engagement at the level of the mechanism that channels the relationship between the social layer of communication and the individual’s capacity for reorganizing mental processes. Attempts to intervene at the level of non-resilient responses, we suggest, can be of only limited effectiveness. This, we argue, explains the lack of clinical response of patients with BPD features to many traditional psychotherapeutic interventions.

A further informing principle is that the type of functioning associated with many forms of psychopathology might best be understood as an evolutionarily driven form of entrenched adaptation to stimuli from the social environment – often in interaction with genetic propensity [7] – rather than as a mere deficit. It is this adaptive imperative that underpins the enduring quality that is central to definitions of PD. The ‘borderline mind’, and related severe problems with social communication typically observed in what we commonly refer to as ‘personality pathology’, may therefore best be understood as a socially triggered outcome, a learned expectation about cultural context. Hence, while the processes we describe in this paper may be implicated in most, if not all, types of psychopathology, we consider severe PD, and BPD in particular, to be prototypical of the type of social communication problems that we now see as lying at the root of vulnerability for severe psychopathology.

Finally, in terms of clinical implications, we will indicate how this change in perspective drives a shift in clinical focus beyond the consulting room to the wider social systems that can promote resilience.

In the first part of this paper we review emerging evidence that a general psychopathology (or ‘p’) factor underlying psychopathology provides a comprehensive explanation for the extensive comorbidity among disorders, as well as many of the other features of individuals who we traditionally consider to be ‘hard to reach’. We then argue that this p factor should not be primarily seen in terms of the presence of specific vulnerability factors (although these may well play an important role, and may be primarily responsible for the phenomenological heterogeneity observed among and within disorders), but in terms of the absence of resilience. We outline the recently formulated comprehensive positive appraisal style theory of resilience (PASTOR), and apply it to BPD as the prototype of disorders characterized by the absence of resilience. We argue that the absence of resilience in BPD results from an inflexibility in the human capacity for social communication, and in problems with recalibrating the mind in the face of adverse experiences in interaction with others in particular.

In the second part of this paper, we will relate this lack of social communicative flexibility to impairments in epistemic trust from an evolutionary and developmental psychopathology perspective, and discuss the clinical implications of this shift in our views.

### A general factor in psychopathology

Our starting point is the challenge presented to the traditional taxonomic structure of psychopathology by comorbidity (concurrent and sequential over time), recurrence and the unwieldy proliferation of diagnostic disorders. In our opinion, this challenge has been compellingly met by the suggestion that there is a general factor of psychopathology – in the words of Caspi and colleagues, ‘one underlying dimension that summarized individuals’ propensity to develop any and all forms of common psychopathologies’ ([3], p. 131). In their analysis of the Dunedin longitudinal study, Caspi et al. examined the structure of psychopathology from adolescence to mid-life, considering dimensionality, persistence, co-occurrence and sequential comorbidity. They found that vulnerability to mental disorder was more convincingly described by one general psychopathology factor – labelled the ‘p’ (for pathology) factor – than by three high-order (spectral) factors (internalizing, externalizing and thought disorder). A higher p factor score was associated with ‘more life impairment, greater familiality, worse developmental histories, and more compromised early-life brain function’ ([3], p. 131). In the meantime, several studies have replicated this higher-order p factor [8–11]. Importantly, the p factor concept may thus also explain why discovering isolated causes, consequences or biomarkers and specific, tailored treatments for psychiatric disorders has proved so elusive for the field [3].

This work on a general factor of psychopathology has recently also been extended to childhood and adolescence. A longitudinal study of 2450 girls aged 5–11 years, for instance, has further indicated the criterion validity of the p factor construct, and found it a significantly better fit than a correlated two-factor (internalizing and externalizing) model [9]. These findings weaken the argument that the p factor is a statistical artefact and reinforce the importance of further consideration of what the p factor might substantively represent [9]. In a large (*n* = 23,477) community-based sample aged 11–13.5 years, Patalay et al. investigated the traditional two-factor (internalizing and externalizing) model and a bi-factor model with a general psychopathology higher-order model [12]. Both models were found to fit the data well; however, the general psychopathology however better predicted future psychopathology and academic attainment 3 years from the time of original assessment; with individuals with high p scores being 10 times as likely to have diagnosable disorder 3 years from assessment than individuals with lower p scores (see also [8]).

More specifically in relation to PDs, Sharp and colleagues have considered the question of whether a general factor for psychopathology exists in the context of PD diagnosis [13]. In a series of exploratory factor analyses based on a sample of 966 inpatients, only four of the six PDs (avoidant, schizotypal, narcissistic, and antisocial) examined formed factors with 75% of the criteria that mark their respective factors. Half the obsessive-compulsive PD criteria loaded with the narcissistic PD criteria, and the other half split across two other factors. However, Sharp et al. found that (a) a BPD factor included primary loadings from just over half (55.6%) of the BPD items, of which three had notable cross-loadings, each on a different factor; (b) nearly half (44.4%) of BPD items loaded most strongly on three non-BPD factors (although two had notable cross-loadings on the BPD factor); and (c) the BPD factor was also marked by a narcissistic PD item and had notable additional cross-loadings by other narcissistic as well as avoidant and schizotypal PD items. In the same study, Sharp et al. evaluated a bi-factor model of PD pathology in which a general factor and several specific factors of personality pathology account for the covariance among PD criteria. In the bi-factor model, it was found that all BPD criteria loaded only on to the general factor. Other PDs loaded either on to both the general and a specific factor or largely only on to a specific factor. The implication of this is that BPD criteria may capture the core of personality pathology, or may be most representative of all PDs. To compound more widely the salient status of BP traits, Caspi et al., in their work on the p factor, found that in terms of personality information, individuals who scored highly on the general psychopathology scale were characterized by ‘three traits that compromise processes by which people maintain stability – low Agreeableness, low Conscientiousness and high Neuroticism; that is, high-p individuals experience difficulties in regulation/control when dealing with others, the environment and the self’ ([3], p. 131). Such a profile appears to capture the core features of BPD – emotion dysregulation, impulsivity and social dysfunction – and speaks to trait profile approaches to PD [14]. Yet, to claim that such a profile in itself in some sense explains the developmental and life-course forecast that comes from ‘p’ obviously risks approaching circularity.

The question that then remains is: what is the meaning of the general psychopathology factor at the level of mental mechanisms? Currently, we can only speculate about the nature of this generic aetiological factor, but one association to be investigated may be childhood maltreatment. Studies indeed suggest that maltreatment, like p, increases the chance of most types of mental illness in adulthood [15] and worsens the course of mental illness [16]. It has been recently suggested that childhood maltreatment may be an ecophenotype associated with an earlier age at onset of psychopathology, greater symptom severity, higher levels of comorbidity, greater risk for suicide and, importantly, a poorer response to treatment [17].

In our opinion, research findings on maltreatment, although still too narrow, indeed point the way to understanding some of the mechanisms underlying the association between the p-factor and vulnerability to (severe) psychopathology This emphasis on the role of adversity should not be associated with a narrowly environmental position on the relationship between adversity and BPD. Such a position would stand counter to growing evidence for a genetic determinant of BPD. Research showing the familial nature of BPD [18, 19], and classical twin studies that place heritability of BPD at around 40–50% [20–23], have been borne out further by more complex behaviour–genetic models that take into account siblings, spouses and twins [24]. Although a genetic anomaly associated with BPD has not so far been identified, it appears that an endophenotype for the disorder may be recognized. For example, impulsive aggression and suicidal behaviour have been linked to the tryptophan hydroxylase (TPH) gene, and patients with BPD have a higher frequency of two out of eight polymorphisms in one of the two known isoforms of the TPH gene [25].

Impulsive aggression has also been connected with reduced serotonergic responsiveness and the inefficient (short or ‘s’) allele of 5-HTTLPR. This has been identified in patients with BPD [26] in some but not all accounts (e.g. [27]). There are suggestions that the s allele marks a vulnerability to stressful life events [28] on the one hand, and the positive influence of maternal sensitivity [29] on the other. Accumulating evidence supports the view that the s allele, in combination with secure attachment, increases agreeable yet autonomous social behaviour in adolescents [30]. In the context of attachment insecurity, this polymorphism is linked with poor self-regulation [31] and impulsiveness [30]. The implication may be that the s allele increases social sensitivity, making a child both more and less prosocial in response to different environmental stimuli.

Furthermore, the methylation of certain genes could mediate the long-term effects of adversity [32]. The glucocorticoid receptor gene promoter, for instance, has been shown to be more methylated in samples of brain tissue of individuals who had experienced adversity and suicide [33]. The methylation of NR3C1 is associated with severity of maltreatment from DNA samples collected from peripheral blood leucocytes in bipolar disorder [34] and also in BPD [35]. In general, inherited differences in specific genes thus may moderate the effects of adversity and determine who is more resilient [36].

Interactional models of biological vulnerability combined with psychosocial risks are therefore being increasingly considered in relation to BPD (e.g. [37, 38]). The emphasis placed on social adversity in this paper should not be regarded as a statement of the exclusive pre-eminence of the environment in understanding the developmental origins of PD. Rather, the assumption that should be understood to underpin our discussion of the role of maltreatment and adversity is that such experiences in individuals who are biologically susceptible (and there may be different genetic routes that lead to this susceptibility) cumulatively strain the viability of resilience and, as we shall demonstrate, epistemic trust.

### BPD as a limitation of psychological resilience

In further clarifying the relationship between BPD and the p factor, Kalisch and colleagues’ [6] conceptual framework for the neurobiology of resilience is enlightening. Kalisch et al. [6] argue that psychological resilience is not an absence of disease processes, but a reflection of the work of active, biologically based mechanisms. In considering the relationship between PD and adversity, we have similarly tended to focus on identifying the characteristics of the patient who is experiencing mental health difficulties rather than attempting to delineate the competencies or capacities of the person who has remained functional and free of disorder despite substantial hardship. In fact, studies suggest that only a minority of individuals develop persisting trauma-related pathology as a result of experiencing or witnessing a single extreme or life-threatening event (e.g. Type I trauma). The majority of people have a remarkable capacity for resilience when faced with such events [39, 40].

Rather than searching for the clinical indicators of a transdiagnostic concept such as p, we may be wiser conceptualizing p as an indication of the absence of resilience and focusing on identifying mechanisms that ‘normally’ protect individuals from harsh conditions. Perhaps p may be more appropriately considered as pointing to protection (or rather the absence of protection).

Resilience has always been an important theme in discourse on mental health [41, 42] but recent concerns about healthcare costs have led to the concept increasingly occupying centre stage [43]. Work on the topic covers myriad different factors and explanations associated with psychological resilience, such as living in a stable and comfortable neighbourhood, family resources and family support, participating in community sporting or extracurricular activities, racial or gender socialization, being securely attached, being able to regulate one’s emotions, exposure to a sensitive style of parenting, or genetic factors. Many of these factors overlap conceptually as well as statistically. They are not explanations for resilience, but rather factors that predict the activation of psychological or biological mechanisms that produce resilience (the absence of pathology in the presence of adversity) as an outcome. Sadly, this conceptual clarity is often lacking in writings about resilience, especially those that concern interventions aimed at its promotion.

The diverse accounts of resilience, often advanced at radically different levels of explanation – from socioeconomic through to genetic – can be unified within the positive appraisal style theory of resilience (PASTOR) conceptual framework presented by Kalisch et al. [6]. According to this formulation, the process underlying resilience is driven by top-down processes in the form of the appraisal that is made of a stressful stimulus. The external and social factors that have been associated with resilience (such as social support or a secure attachment history) affect resilience either directly or indirectly in that they shape the individual’s appraisal approach, or minimize exposure to stressors. This is not to deny the role of socio-environmental factors in determining an individual’s resilience, or to deny the importance of interventions at a social or community level; it is to suggest that the mechanism by which these distal social factors affect individual resilience is via their impact on the individual’s appraisal style.

### Resilience and reappraisal

The appraisal theory of resilience is based on a specific understanding of the nature of higher-order cognition [44]. The theory is that the resilience process is as follows: a potentially stressful stimulus is perceived and mentally represented by the individual. The mental representation is then appraised using higher-order cognition, understood in terms of an ensemble of psychological mechanisms and phenomena, including executive function, attention, general intelligence and self-awareness. This in turn determines the emotional response of the individual – their resilience.

We consider this an important perspective but a narrow interpretation of what may be considered higher-order cognition. The outputs of neural processing intrinsically depend on the processing units that take input from the output of other units, perform specific functions, and generate output that in turn becomes the input of other processes. In most models of brain function, any psychological capacity is underpinned by a large number of such hypothetical processes [45, 46]. In this context, the nature of the organization of processing units, or indeed the system that determines their relative activation, may be either a simple function of the efficiency of processing or, within a hierarchical system, determined by the functioning of a higher-order system. The higher-order meta-system monitors the performance of lower-order systems to ensure optimal performance within a particular context. These components of higher-order cognition are what constitute the core of a normal wakeful and wilful mind in the process of conscious perception, imagination, decision-making and action planning. These functions, taken together, create an opportunity for the internal reorganization of neural structures within the human brain. A consistently ‘self-observing’ process, which monitors the quality of outcome of neural processing units, enables the individual to reorganize the way neural structures subserve cognitive function. Mentalizing is a key facet of this self-observational process, and the extent to which intentionality fulfils expected behavioural outcomes is a critical indicator of the efficiency of neural processing and guides the way information processing is organized within available pertinent neural units. We assume that an efficiently functioning human brain representing a resilient system achieves such robustness because mentalizing provides a clear window on the efficiency of brain functioning. Multiple processing units cover similar functions in the brain. Some units, being more efficient than others, are more likely to be providers of output that is taken forward to other units. But circumstances change, and demands for adaption may reverse the hierarchy of efficient functioning of these processing units. Resilience is the appropriate appraisal and monitoring of the external social environment and internal functioning of processing units. Thus, as we will explain in more detail in Part 2 of this paper, higher-order cognition is the developmental capacity, based on early relationships and constantly renewed in changing social contexts, to appraise the efficiency of functioning, which in essence is intersubjective in its nature. The capacity to anticipate the reaction of another person, to regulate attention or to implement action plans are all shaped by the overarching need for survival in the context of social interaction. A failure of resilience arises when the individual is unable to change processing systems in a sufficiently flexible manner to maintain optimum outcome despite changed circumstances. When an individual cannot disengage a processing system that is no longer appropriate to the task – for example, a child whose perfectionistic attitudes serve them well during a period of knowledge acquisition and relatively simple tasks, but cause great problems when task complexity has increased to a point where perfection is impossible – the lack of flexibility is what creates vulnerability. Insensitivity does not create risk; the sensitivity of higher-order cognition is what provides protection through the appropriate appraisal of the functioning of neural structures relative to the environment. This is how the resilient brain functions; it is not a model that skirts reification – it is a description of our assumptions of the nature of brain function.

Higher-order cognition appears to be more flexible within the brain than other, more specialized modal forms of cognition such as basic vision and hearing. For example, brains are able to preserve core aspects of the functional architecture of the information processing that sustains higher-order cognition in spite of substantial structural damage [47]. Higher-order cognition is a form of information processing, therefore, that does not completely rely on one single, static or fixed set of specialized brain regions and anatomical connections, within certain limits of course. It works by exploiting available neural resources and possible routes between them; it seems to use degenerated and pluripotent brain systems flexibly, enabling higher-order cognition to emerge as one of the most robust brain functions. In that sense, the mind does not exist in one physical location within the brain; rather, it is an abstraction, or code, and the brain is the code interpreter. Basic consciousness – the mechanism for the resiliency of cognitive and control systems – is thought to have evolved to be maximally resilient itself: ‘consciousness itself can be interpreted as a general algorithm for resilience selected by evolution’ ([47], p.22). This decoupling of higher-order cognition from a single location appears to be highly adaptive: its relatively abstract and algorithmic nature makes it more robust in the face of any localized damage or degeneration within the brain.

The algorithmic quality of consciousness may be regarded as a pinnacle of human evolution, but this should not detract from its highly pragmatic, adaptive purposes. This resilient framework is an essential condition for functioning autonomy and the capacity to adapt to the world’s demands – particularly the highly complex demands of the human social world. As Paradiso and Rudrauf [48] have argued in their article on social cognition and social neuroscience, tellingly entitled ‘Struggle for life, struggle for love and recognition: the neglected self in social cognitive neuroscience’, the self, self-awareness and intersubjectivity are integral to social cognitions and actions. As described above, the appropriate functioning of higher-order cognition crucially depends on appropriate judgements about social contexts. In this sense, social cognition is part of the mechanism of higher-order cognition, although social cognition itself is made up of a set processes that are monitored by the metacognitive evaluations that higher-order cognition performs: as in any feedback system, there is an inherent circularity in this conceptualization. This is inevitable given that we are describing the extent to which a system is capable of reorganizing its own functioning. Similarly, the modes of operationalizing the self and the identification of self-awareness are strongly shaped by developmental contributions from the social environment – parents, sibling, peers and significant others. In other words, the abstract algorithm that creates personal consciousness cannot be separated from social interactions. This is what the algorithm was developed for, and what further shapes the algorithm of the self and its ongoing relationship with the outside world.

Although there are many factors at work in contributing to resilience, Kalisch describes the three underpinning appraisal mechanisms that determine resilient behaviour and responses [6], as follows:

1. *Positive situation classification*. This refers to the manner of immediate appraisal of a situation at the moment of encountering it (e.g. ‘What is the person approaching me carrying in their hand?’). In the case of an insignificant threat, a positive appraisal style enables the individual to view it in a manageable perspective. Clearly, in the context of an adverse event, a negative appraisal and stress response are called for. In such situations, resilience can be subsequently promoted through the second and third forms of appraisal.
2. *The retrospective reappraisal of threat*. Whether a traumatic event results in post-traumatic stress disorder, for example, is dependent on how it is retrospectively reappraised [49, 50]. This, as Kalisch et al. describe it, ‘shifts the emphasis from the external situation (or changes in the situation) to the individual’s ability to flexibly adjust current negative appraisal or to implement new, more positive appraisals and then to maintain those appraisals. Both processes have to occur in the face of interference from automatic and uncontrolled negative appraisals and the accompanying aversive emotional states’ ([6], p. 14).
3. *Inhibition of retraumatizing triggers*. This mechanism enables the individual to inhibit the threat-associated sensations that might be experienced when remembering a traumatic event and serve to reinforce, perpetuate and generalize the sense of threat.

### BPD and the PASTOR model of resilience

To return to BPD, we can follow the PASTOR model by distinguishing between resilience factors and mechanisms. We suggest that a traditional clinical mistake in the treatment of BPD has been to intervene at the level of resilience factors rather than at the level of appraisal (i.e. mechanisms) – this in effect means that we have been working at the level of correlation rather than causation. In BPD, the appraisal mechanisms are at fault, in large part because of mentalizing difficulties (e.g. in the mistaken appraisal of threat at the moment of its presentation) or a breakdown in epistemic trust, which damages the capacity to relearn different ways of mentalizing – or appraising – situations (i.e. the inability to change our understanding of the threat after the event). The outcome is the lack of resilience that is highly characteristic of BPD, regardless of its clinical presentation.

#### BPD and positive situation appraisal

Mentalizing has an interpretive role and allows us to explain and predict behaviour; in this sense it also has a social regulatory role [51]. Behaviour can be produced by rational interactions among beliefs and desires, which, when interpreted (appraised) according to specific culturally determined expectations, generate meaning (a meaning assigned to the observed action) in terms of putative mental states that could have engendered the perceived behaviours. Therefore, for our behaviour to be socially meaningful (predictable), it can and should obey these same conventions. Frequent behavioural deviations from these expectations may be considered as being core to PD. This is confusing and stressful for the observer because the normal process of reconstructing mental states from actions is disrupted.

The great importance of this process of meaning generation has been powerfully illustrated by studies in which participants were led to believe that deterministic neurological processes, rather than mental states, control behaviour: in other words, they were discouraged from believing in free will. Introducing an abstract disbelief in free will led to an observed weakening of neural signals associated with readiness planning; subjects became less prepared to act voluntarily [52]. Setting up a deterministic neurological bias also appeared to ‘free’ individuals from a sense of personal responsibility and generated more antisocial cheating and aggression [53].

If mentalizing is assumed to have such an interpretative and regulatory role, then individuals with BPD who have limited capacity to exercise this regulative function are at least partially deprived of the appraisal processes needed to reduce the stress of any social experience. This leaves them at times confused and vulnerable in both the interpretation and the convention-governed expression of mental states in behaviour. To put it plainly, they are frequently puzzled by others’ actions, and equally find themselves victims of misattributions by others. There is ample clinical evidence of limitations of appraisal in BPD (for examples, see [54–57]) although undoubtedly, as would be predicted by the p factor model, they are by no means the only clinical group to show concerning limitations in this area. Poor appraisal may be more severe and pervasive in BPD than, for instance, in major depressive disorder or generalized anxiety disorder without PD comorbidity.

Individuals with BPD tend to be very prone to automatic, non-reflective mentalizing; they often base their inferences on the immediate exterior features of others, and rely on affective rather than cognitive mentalizing. This has clear implications for the style in which they are likely to appraise social situations. As a result of their mentalizing tendencies, individuals with BPD tend to appraise situations and read others’ expressions quite quickly: they may show a hypersensitivity to facial expressions [58, 59] and higher-than-normal sensitivity to non-verbal communication [60, 61]. For example, individuals with BPD have been found to outperform non-BPD comparisons on the Reading the Mind in the Eyes Test [62] or to be at least as good as normal controls on the same test [56, 63]. However, this emphasis on external and immediate cues in appraisal situations is accompanied by difficulties in making more reflective judgements based on what might be going on inside people’s minds – so, for example, individuals with BPD tend to perform more poorly in social exchange tasks [55, 64]. They have also been found to be more likely to view characters/behaviours as negative or aggressive [65]; to have an impaired view of neutral faces in the context of anger or disgust [66]; and to react with hostility to neutral social interactions [67] – all suggestive of the negative appraisal style described by Kalisch and colleagues [6]. The emphasis on affective mentalizing also results in a heightened sensitivity to emotional cues [59], especially in cases of anger and fear [68, 69]. Furthermore, unbalanced mentalizing on the self–other dimension can cause individuals with BPD to experience severe difficulties in separating the self from the other [70–73] and to be unduly emotionally affected by others’ affective states. This often leads to the experience of emotional contagion, which has clear implications for social appraisal situations [74, 75]: BPD individuals can feel forced to be rigid and highly controlling in order to maintain a subjective sense of coherence and integrity [76].

The mentalizing profile characteristic of an individual with BPD, in sum, results in an oversensitivity to possibly difficult social interactions (because distortions in mentalizing are more likely to result in mistaken interpretations of others’ behaviour and motivation). In the aftermath of a challenging or stressful interaction, it is difficult for the individual to make sense of, contextualize or put aside potentially upsetting memories of experiences, leaving them more vulnerable to emotional storms. A capacity for explicit, reflective mentalizing in particular serves a dual interpretive (appraisal-strengthening) and self-regulatory role. The absence of this capacity deprives the individual of a fundamental tool in reducing stress.

However, one can see that in certain situations, for example, an emergency milieu characterized by high levels of interpersonal aggression, the heightened and immediate sensitivity and seemingly instinctive and physically charged form of appraisal characteristic of BPD might in fact be adaptive, at least in the short term. In such an environment, extreme vigilance is a potential advantage, and similarly, the ability to form intense emotional relationships quickly might elicit resources or protection. The mentalizing profile associated with BPD and the appraisal style this generates is maladaptive in most stable social contexts, but we postulate that this mentalizing profile may be a response to cues suggestive of an unreliable and potentially threatening social environment. We thus should be wary of seeing apparent dysfunctions of the clinically ‘hard to reach’ as indicative of a deficit or any kind of sub-optimal functioning (as, indeed, we have done previously [77]). We would now consider that what may appear to us as dysfunction is an evolutionarily primed adaptation to specific environmental and social contexts. As a genetically triggered adaptation, the individual is biologically programmed to resist change in a behaviour pattern that signals increased chances of selection. We believe that enduring mental disorders (including BPD) are nested in the context of the evolutionary priorities of the human condition.

#### BPD and retrospective reappraisal

The mentalizing difficulties of BPD patients have also considerable implications for understanding the difficulties with retrospective reappraisal that may undermine resilience. Reappraisal can attenuate ongoing stress responses by appropriately adjusting negative appraisals and/or generating complementary positive appraisals. In strongly aversive situations the stress response is essentially unavoidable: the experience is automatically classified as negative and requires ‘after the event’ changes in the meaning of the stimuli. This is often achieved through reappraisal in terms of the mental states of the protagonists. To retrospectively appraise an event or situation in a way that promotes resilience, an individual needs to be able to reappraise it in a way that involves reflective, cognitive mentalizing. Such reappraisal will often also depend upon a capacity to mentalize the internal states of both the other and the self. In other words, the mentalizing strengths that this form of retrospective reappraisal requires are not congruent with the mentalizing profile typical of BPD, which is characterized by (a) a tendency to focus on the external rather than internal states of others; (b) the dominance of automatic, intuitive mentalizing over controlled, reflective, mentalizing that could help to put the potentially traumatic event into perspective;(c) an imbalance between affect and cognition in favour of the former, leading to self-perpetuating persistence of negative affect; and, finally, (d) difficulties in coherently representing the self independently of the other, undermining the potential to contextualize and make proportionate an event.

The mentalizing model for trauma has reappraisal of physical and psychological experience at its core [78, 79]. Similarly, trauma-focused cognitive-behavioural therapy and other exposure-based therapies (e.g. eye movement desensitization and reprocessing therapy) enhance mentalizing of the trauma experience, creating a second-order representation of the event in terms of greater coherence of the subjective experience of the victim and often also the perpetrator. Patients with BPD have a specific problem in relation to reappraisal proper because they find it challenging to generate second-order representations of mental states that might be modified to constitute more positive reappraisals of experiences or modify and thus mitigate (adjust) negative appraisals. In essence, this lies at the core of Gunderson and Lyons-Ruth’s interpersonal hypersensitivity theory of BPD [80]. Interpersonal hypersensitivity is the likely consequence of a failure of reappraisal following stressful social interactions. In the absence of being able to mentalize in a balanced way, an event or a relationship can be endlessly discussed and dissected in an apparent attempt at reappraisal, but such attempts have an unreal quality. Complicated inferences about mental states are made, but they might have little connection with reality. We term this *pseudomentalizing*, or in extreme, *hypermentalizing*; it is a state of mind that can be clinically misleading in that it may present as a strong attempt at reflection and engagement, but it will ultimately be circular and unproductive. Hypermentalizing of trauma, the failure to move on from it, may be inevitable if individuals cannot reliably access and use social communication that could enable them to resolve or contain the sense of threat associated with a trauma (or if a perceived threat that has been misinterpreted as such, owing to problems in the first resilience mechanism). However, as our understanding of this state of ‘petrification’ has deepened [81], we also have come to recognize that mentalizing is not everything, or rather, that bodily experience has an important role in enabling access to further resilience strategies. This brings us to the importance of inhibition mechanisms.

#### BPD and the interference inhibition mechanism

According to Kalisch et al.’s conceptual framework [6], the final level of appraisal underpinning resilience is an inhibition mechanism based on interference. As mentioned above, a strongly aversive event naturally generates powerful negative appraisal responses. The ability to moderate and regulate such negative responses after the event can further determine the extent to which the event continues to cause difficulties in psychological functioning. This implies the inhibition of conflictive negative appraisals and acting deliberately to interfere with emotional reactions to information processing. The inhibition of negative and disruptive responses through distraction or interference can enable the individual to begin the process of reappraisal proper, allowing a more resilient response to emerge. An individual’s inhibitory capacity may to a large part be a trait-like characteristic, with some genetic basis. However, the extent to which the inhibition mechanism can be overwhelmed and how its restoration can be managed may be malleable to some degree.

Although much has been written on the nature of traumatic experiences, within the view outlined in this paper, an aversive event becomes traumatic in its aftermath when it is accompanied by a sense that one is not accompanied – that one’s mental experience is not shared and the ‘mind is alone’ [78, 82]. Trauma obtains from a primitive, adaptive human terror of isolation. Here, again, we run into the key importance of social referencing to calibrate the mind. In the process of reappraisal, the social referencing provided by being able to access another mind enables us to frame and put into perspective an otherwise overwhelmingly frightening experience. This process, which drives a so-called broaden-and-build cycle [83], is far more available to individuals who are open to the benign social influence of other minds. As outlined in more detail in Part 2 of this paper, those who are able to manifest sufficient levels of epistemic trust to embark on the mutually mentalizing stance that is essential in soliciting other minds in support of one’s own, are therefore more likely to be resilient. The commonly observed vicious cycle of BPD, comorbid trauma and the acute subjective experience of isolation captures the implications of the failure of this inhibition reappraisal mechanism.

Individuals with the diagnosis of BPD have been shown to have serious limitations in their capacity for the inhibition of conflictive negative appraisals and for interfering emotional reactions to information processing. They cannot cognitively inhibit retraumatizing triggers, leaving them vulnerable to threat-associated sensations that might be experienced when remembering a traumatic event, which serve to reinforce the sense of threat. It is not possible for these individuals to access mentalizing if the self is overwhelmed by negative interference that impairs normal cognitive function. This is congruent with the view that emotional dysregulation is the fundamental problem in BPD [84–86]. The idea of a failure of inhibition in BPD also echoes recently reported findings from Koenigsberg et al. concerning the failure of habituation in BPD [87, 88], which may have a genetic basis [89].

We have similarly (albeit not formulated in terms of the failure of interference or habituation) described the phenomenology of the unyielding nature of trauma-linked subjective experience in BPD [90] in terms of alien self experiences that consist of a sense of looming, unmanageable anxieties that cannot be reappraised and contained, as the subjective outcome of incorporating an experience of overwhelming hostility into the self [91]. In this context, the focus is not on the development of this experience but rather how it is so persistently maintained despite intense and persistent efforts at reappraisal. This shift in perspective involves a recognition of the significance of the capacity for inhibition in the treatment of BPD. Individuals who are very poor at mentalizing may require more than cognitive interventions (talking) to bring about the inhibition of the stress response. Interventions may have to relate to the body more directly. We have always had a view that mentalizing was embodied [92], but we have not treated this fact seriously enough. We now see a role for physical activity in strengthening the capacity for inhibition at the same time as helping to restore mentalizing. In clinical terms, we suggest that physical activity has a role in strengthening the capacity for inhibition at the same time as, or as a precursor to, helping to restore mentalizing. Perhaps new areas for developing effective interventions may lie in this direction. For example, if an adolescent cannot communicate, activating interference to permit reappraisal via physical activity may well be more valuable than spending hours attempting to activate mentalizing via talking and reflection. The best initial approach may be a physical one: running with them, and discussing what the running was like. Such a simple focus on the embodied experience can be used to begin to rehearse the most basic principle of responding to and giving space to a stimulus outside the negative responses that normally overwhelm other forms of social cognition.

## Conclusions

Although we still consider attachment and mentalizing to be key in our understanding of personality pathology, and in earlier formulations we have always emphasized the importance of the absence of resilience in BPD, there has been a notable shift in our views on the emergence and nature of BPD. Rather than seeing BPD primarily in terms of the presence of impairments in attachment and mentalizing, we consider the notable absence of resilience and the social communicative inflexibility that seems to underlie this absence as an adaptive strategy that individuals with BPD acquire within a social context where social inflexibility was often the only possible survival strategy and had considerable advantages in the short term.

We will further elaborate on these issues in Part 2 of this paper. Currently we are still faced with an important theoretical dilemma: from where does this absence of positive reappraisal mechanisms stem? How can we understand the inflexibility in social communicative processes in BPD and in all those suffering from serious psychopathology, which seems to render these individuals so ‘hard to reach’? How did this inflexibility develop over time? We believe that the answers to these questions lie in an evolutionarily informed developmental psychopathology account of BPD and related disorders that has considerable implications for prevention and intervention.

## Abbreviations

BPD: Borderline personality disorder
PASTOR: Positive appraisal style theory of resilience
PD: Personality disorder
